# Promoting Resilience and Well-being Through Co-design (The PRIDE Project): Protocol for the Development and Preliminary Evaluation of a Prototype Resilience-Based Intervention for Sexual and Gender Minority Youth

**DOI:** 10.2196/31036

**Published:** 2022-02-01

**Authors:** Mathijs F G Lucassen, Rajvinder Samra, Katharine A Rimes, Katherine E Brown, Louise M Wallace

**Affiliations:** 1 Department of Health and Social Care The Open University Milton Keynes United Kingdom; 2 Institute of Psychiatry, Psychology and Neuroscience King's College London London United Kingdom; 3 Department of Psychology, Sport and Geography University of Hertfordshire Hatfield United Kingdom

**Keywords:** LGBT, e-therapy, depression, adolescent, youth, online, sexuality, gender, resilience, public health

## Abstract

**Background:**

Sexual and gender minority youth (SGMY) are at an increased risk of a range of mental health problems. However, few evidence-informed interventions have been developed specifically to support their mental well-being. Interventions that are evidence-informed for the general population and are fine-tuned specifically with SGMY in mind proffer considerable potential. A particular opportunity lies in the delivery of engaging interventions on the web, where the focus is on enhancing the coping skills and building the resilience of SGMY, in a way that is directly relevant to their experiences. On the basis of earlier work related to an intervention called Rainbow SPARX (Smart, Positive, Active, Realistic, X-factor thoughts), we seek to create a new resource, especially for SGMY in the United Kingdom.

**Objective:**

This project has 3 main objectives. First, together with SGMY as well as key adult experts, we aim to co-design a media-rich evidence-informed web-based SGMY well-being prototype toolkit aimed at those aged between 13 and 19 years. Second, we will explore how the web-based toolkit can be used within public health systems in the United Kingdom by SGMY and potentially other relevant stakeholders. Third, we aim to conduct a preliminary evaluation of the toolkit, which will inform the design of a future effectiveness study.

**Methods:**

The first objective will be met by conducting the following: approximately 10 interviews with SGMY and 15 interviews with adult experts, a scoping review of studies focused on psychosocial coping strategies for SGMY, and co-design workshops with approximately 20 SGMY, which will inform the creation of the prototype toolkit. The second objective will be met by carrying out interviews with approximately 5 selected adult experts and 10 SGMY to explore how the toolkit can be best used and to determine the parameters and user-generated standards for a future effectiveness trial. The final objective will be met with a small-scale process evaluation, using the think out loud methodology, conducted with approximately 10 SGMY.

**Results:**

The study commenced on September 1, 2021, and data gathering for phase 1 began in October 2021.

**Conclusions:**

A considerable body of work has described the issues faced by the SGMY. However, there is a dearth of research seeking to develop interventions for SGMY so that they can thrive. This project aims to co-design such an intervention.

**Trial Registration:**

Research Registry Reference researchregistry6815; https://www.researchregistry.com/browse-the-registry#home/registrationdetails/609e81bda4a706001c94b63a/

**International Registered Report Identifier (IRRID):**

PRR1-10.2196/31036

## Introduction

### Background

It has been estimated that up to 10% of the adolescent population are sexual and gender minority youth (SGMY), as determined by the results of a range of population-based samples [1,2]. Despite rapid social progress, SGMY often experience abuse, bullying, and victimization. For example, the United Kingdom’s nationally representative Millennium Cohort Study of almost 10,000 adolescents reported that sexual minority youth had twice the odds of being verbally abused (odds ratio 2.25) and physically assaulted (odds ratio 2.15) in the past year compared with their heterosexual peers [3]. Less has been documented regarding the mistreatment of gender minority youth, which draws on population-based data. However, reports that do exist highlight a particularly disturbing picture of abuse and discrimination [4,5]. Abuse, mistreatment, and socially hostile environments are thought to be key drivers that impact the mental health of SGMY [6,7]. This, by extension, places them at a considerable risk of depression and other mental health problems. For instance, a meta-analysis of population-based studies (predominantly from the United States) that included 165,380 adolescents highlighted that sexual minority youth had almost 3 times the odds of depressive symptoms and depressive disorder (odds ratio 2.94) compared with their heterosexual peers [1]. Population-based estimates of depression among gender minority youth are even more concerning, with a nationally representative study of secondary school students in New Zealand reporting that transgender adolescents had almost 6 times the odds of clinically significant depressive symptoms (odds ratio 5.7) compared with their peers who were not transgender (ie, were cisgender) [8]. Therefore, a pressing public health challenge is addressing the adverse effects of social violence experienced by these adolescents in the context of a heteropatriarchal society, which places them at greater risk of mental ill-health.

Typically, SGMY cannot simply leave the harmful social environments that impact their mental health owing to practical constraints, including their economic dependence on their families. Many are geographically isolated away from the SGMY supports clustered in cities, and most will not have parents who are sexual and gender minority (SGM) individuals. Further exacerbating these challenges is evidence that SGMY appear to be *coming out* at a younger age [9] when they will have had fewer opportunities to develop effective strategies to cope with stigma processes [7]. Hence, there is an urgent need for widely accessible and targeted help to assist these adolescents in developing the best possible skills to thrive. This need has been reinforced in policy documents. For example, public health policy in England prioritizes the “reduced development and exacerbation of mental health problems, including among high-risk groups and children and adolescents” [10].

There are published studies on strategies to improve harmful social environments, including student-led clubs for SGMY and antibullying interventions delivered face-to-face by SGMY organizations in secondary schools (eg, [11,12]). However, these interventions often miss those who most need them [13]. This includes SGMY in the most challenging environments, not because they are necessarily hard to reach but, as seen with other underserved populations, they will be easy to neglect [13]. As a result, the initiatives to improve the social milieu for SGMY tend to be deployed in environments already supportive of SGMY.

Although SGM people have previously been identified as a *high-risk* population in terms of suicide by the UK government [14], few evidence-informed interventions have been developed specifically for SGMY. In addition, SGM individuals are poorly served by the options available to them in terms of effective *mainstream* interventions [15-17], with SGMY frequently perceiving health care providers as unhelpful [18]. To date, most research in the field has focused on describing the issues SGMY face and not on possible solutions [19,20]. It is already known that SGMY frequently seek informal support on the web [18], but 2 systematic reviews focused on psychosocial treatments published in 2018 and 2019 identified only a single evaluated digital tool to support the mental well-being of SGMY [19,20], and this tool, *Rainbow SPARX* (Smart, Positive, Active, Realistic, X-factor thoughts), was developed in New Zealand [21]. A third more inclusive systematic review was published in 2020 [22]. This review sought to provide an overview of all digital health interventions that aimed to address the mental, physical, or sexual health–related concerns of SGMY. Most of the interventions identified (17 of the 24) targeted the management or risk reduction of sexually transmitted infections, with only 5 interventions primarily targeting mental health–related problems [22]. Of these 5 interventions, 1 focused on drug abuse prevention, 2 targeted nonspecific aspects of psychological well-being using YouTube videos and expressive writing techniques, and 2 targeted internalizing symptoms or problems. The 2 interventions focused on internalizing symptoms were Rainbow SPARX and TODAY! [23]. The TODAY! intervention, from the United States, is an app that offers young sexual minority men techniques that they can use to manage their symptoms of depression and anxiety. It has been subjected to usability testing, and the qualitative data gathered from this testing will be used to inform the later stages of this intervention’s development [23]. The paucity of identified digital tools for SGMY is somewhat surprising given that a UK Department of Health commissioned report has highlighted SGMY’s strong preference to access help on the web. In this report, 82.3% (n=572 SGMY) of participants indicated that they would be *likely* or *very likely* to choose help in this format. This was followed by a preference for face-to-face assistance (n=355 SGMY, 51.1%) and then mobile (eg, SMS text messaging) forms of support (n=297 SGMY, 43.2%) [18].

Evidence-informed web-based interventions are rarely made available to young people, for instance Rainbow SPARX has not been used outside of a research context. This intervention was co-designed together with SGMY [24] and evaluated in a mixed methods open trial [21,25]. Most recently, a further intervention for SGMY has been delivered digitally for young people (aged 14-29 years) in Canada, also within a research context [26]. The intervention *AFFIRM Online* consists of 8 sessions facilitated by an SGM clinician and is provided on the web in a synchronous manner [26]. Arguably, requiring SGM clinician facilitation limits the intervention’s ability to potentially support large numbers of SGMY. Therefore, interventions that are evidence-informed, widely accessible, and fine-tuned with SGMY in mind (eg, with strategies that assist them in managing SGM stigma and victimization) offer considerable potential. This is especially so if delivered on the web as self-help, while harnessing innovations in multimedia design and with a focus on enhancing coping skills.

### Objectives

This project has three main objectives:

1. To co-design a media-rich evidence-informed web-based SGMY well-being prototype toolkit together with SGMY as well as key adult experts
2. To explore how the web-based toolkit can be used within public health systems in the United Kingdom by SGMY, and potentially other relevant stakeholders
3. To conduct a preliminary evaluation of the toolkit, which will inform the design of a future effectiveness study

## Methods

### Target Population and Outcomes

We have decided to cocreate a toolkit for SGMY as a combined group and not a separate resource for either sexual minority youth or gender minority youth. We have come to this decision based on our experiences of attempting to initially focus exclusively on sexual minority young people for Rainbow SPARX-related work, but where we eventually found a focus on SGMY was optimal. In particular, during the recruitment phase of the open trial of Rainbow SPARX, gender minority youth also wanted to participate (and were included) [21]. Moreover, during the trial period, some participants’ gender and sexual identities were not static and evolved (understandably given that they were all adolescents). Therefore, we think that a focus on SGMY as a whole is preferable, given the developmental factors and so that we are inclusive of young people who will be fluid, questioning, or unsure of their sexual identity or gender identity. As such, we believe that an inclusive and combined approach will present the best opportunity to reduce the barriers and enhance inclusion in this research.

The target outcome for the toolkit is the improved overall well-being of SGMY. At this stage, we have deliberately not stipulated anything more detailed in terms of outcomes, as the outcomes will be confirmed during the CONCEPT stage (see Table 1 for a summary of the project’s stages). For instance, during the IDENTIFY to POSITION stages of the project, SGMY may highlight a preference for a reduced focus on managing symptoms of depression, which has been a key outcome or focus of digital interventions for SGMY to date (eg, for Rainbow SPARX [21], TODAY! [23], and AFFIRM Online [26]). Instead, SGMY may recommend, for example, that behavioral strategies to better handle peer bullying be a major focus of the toolkit. To determine what SGMY believe should be the specific target outcomes, we will use a modified nominal group technique [27]. For instance, SGMY will be asked to rank the importance of certain features or outcomes based on responses using a Likert scale (eg, a feature or outcome is ranked from 1=very important to 5=not at all important). This technique has been successfully used in focus groups or workshops (eg, [28]), as well as prior related work where young people (aged 16-25 years) codeveloped user-generated quality standards for youth mental health services in primary health care settings [29].

**Table 1:** Overview of the study’s stages^a^.

	IDENTIFY	DEFINE	POSITION	CONCEPT	CREATE	USE
Methods which enable the active participation of adolescents	*How do SGMY*^b^*see the biggest systemic and environmental problems? *Based on: phase 1 interviews with SGMY & data from published studies	*How do the problems [from IDENTIFY] manifest in the lives of SGMY & how do they cope? *Phase 1 interviews with SGMY [& ‘card sorting’ hierarchy of problems]	*How should the evidence-informed coping strategies be communicated to SGMY?* Co-design workshop 1 with SGMY [to include creating scenarios & discussing ‘look & feel’]	*What would motivate SGMY to use the online toolkit?* Co-design workshop 1 [to include creating storyboards & generating design ideas]	*How should it/could it be improved?* Co-design workshop 2 [to include providing corrective feedback on rough cuts of the audio-visual materials & toolkit prototypes]	*Is the online toolkit successful from the perspective of SGMY?* In-depth process evaluation [to include interviews with SGMY with no prior knowledge of the toolkit]
Evidence-informed research activities	*What do adult experts see as the biggest environmental problems?* Consultations and interviews with adult experts	*Literature review.* Scoping review to determine evidence-informed coping strategies for SGMY	*Determine the best online & creative techniques.* Confirm the behavioral theories most relevant to the overall project	*Stakeholder consultation with adult experts.* What should be the process, impact, and outcome indicators?	*Develop protocols for possible future randomised controlled trial.* Build and test data collection within toolkit	*Determine toolkit’s applicability & evaluate toolkit.* Interviews with adult experts to establish toolkit’s use

^a^Stages as informed by the earlier work of Hagen and colleagues [30].

^b^SGMY: sexual and gender minority youth.

### Participants and Their Recruitment

The participants will consist of SGMY and key adult expert stakeholders.

#### SGMY Participants

We will recruit approximately 40 SGMY aged between 13 and 19 years residing in the United Kingdom. SGMY will be recruited via youth workers, including those based at the SGMY centers who have already expressed an interest in being involved in this study. These centers are based in a range of geographic locations, including a large urban area where the SGMY using their services are diverse in terms of their ethnicity, socioeconomic status, sexual identity (eg, they may identify as lesbian, gay, bisexual, or *questioning*), and gender identity. As a result, we expect to have cisgender male, cisgender female, and transgender participants (eg, transgender women, transgender men, and nonbinary young people) participating.

#### Key Adult Expert Participants

These participants (all resident in the United Kingdom) will consist of the following:

- Health and social care professionals (eg, SGM youth workers, public health practitioners, and other health care providers)
- Other publicly employed professionals with an interest in SGMY mistreatment prevention (eg, specialist teachers, experts on bullying prevention, and SGM police officers)
- Professionals who commission health and social care services (eg, staff working in a clinical commissioning group or local authority)
- Parents of adolescents interested in supporting the well-being of SGMY

Approximately 20 adult participants will be recruited via organizations that have endorsed this study and have agreed to support its implementation (eg, SGMY centers, 2 county councils in Southern England, and a center for police and policing research). The parents will be recruited via the networks of the Promoting Resilience and Well-being Through Co-design (PRIDE) team members (eg, support groups for SGM staff and parent groups hosted by a university). We will recruit a range of adult experts, including those who are likely to be SGM themselves. We believe that adult participants who are heterosexual and cisgender will also contribute meaningfully to the study, first, because of their roles (eg, there is a fairly limited number of health and social care commissioners) and, second, because of their expertise and practical experience (eg, some practitioners are highly skilled at working with SGMY and are heterosexual and cisgender).

### Toolkit

It is envisaged that the toolkit will have the following characteristics:

- Be accessible (ie, conform to Web Content Accessibility Guidelines 2.1) and the website will be responsive by assuming a different structure as needed for desktop, mobile, and device use;
- Have embedded standardized assessments and measures (see the Measures section for further details);
- Involve a degree of interactivity, which might include gamification elements (eg, a *points-based system* that could be amassed as the user attempts to support the SGMY *characters* in the most helpful way possible) as gamification is thought to improve engagement with mental health interventions [31]; and
- Include short dramatizations in video format as part of relatable vignettes that are embedded into the toolkit. For example, it is anticipated that 3 vignettes will be created, each focused on a specific challenge, such as X who is a professional and SGMY ally struggling to support a young person being bullied because of being bisexual, Y who is preparing to tell their parents they are gender nonbinary, and Z who is being pressured into attending prayer-based *therapy* (to *become straight*).

To maintain the confidentiality and safety of participants, the proposed web-based toolkit and the associated video material will be filmed with actors who constitute the *characters*. Nonetheless, attempts will be made to situate filmed scenes in realistic environments to promote the engagement and uptake of materials.

**Figure 1:**
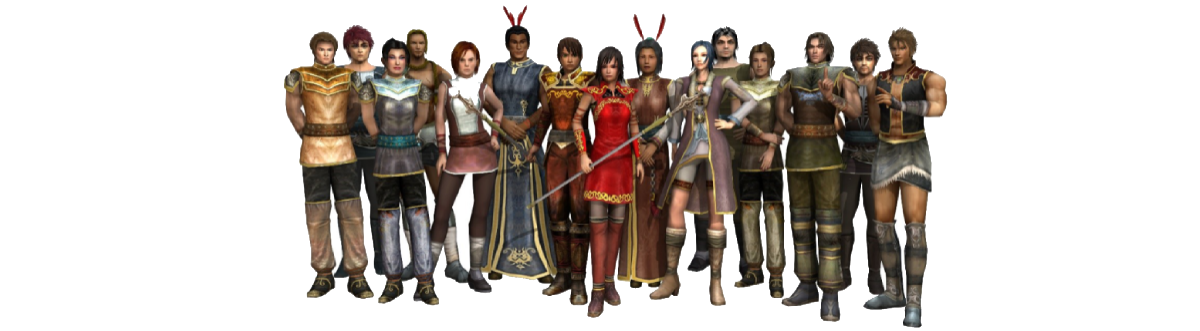
Rainbow SPARX (Smart, Positive, Active, Realistic, X-factor thoughts) characters (the female character dressed in red holding a staff and the male character immediately beside her are the avatars).

The toolkit will also build upon and advance aspects of the work already conducted in relation to Rainbow SPARX [21,25]. This 7-level form of computerized cognitive behavioral therapy (CBT) was designed to treat depression in SGMY and was based on the original version of SPARX, a serious game-based intervention [32]. The differences between SPARX and Rainbow SPARX are mostly script-related and account for 5.9% of the overall program script (ie, the minigames, characters, and weekly homework tasks remained the same) [21]. The main version of SPARX has been enhanced and updated for web-based delivery, and it is currently delivered free of charge in New Zealand. However, Rainbow SPARX has not been updated since 2009, nor has it been used outside of a research context. Prior research conducted with SGMY has reinforced issues or challenges to do with Rainbow SPARX, which is why we are developing a new bespoke toolkit specifically for SGMY in the United Kingdom. In particular, user feedback from SGMY about Rainbow SPARX has reinforced that substantial changes are required [25,33], and it has not been possible to address all the key issues to ensure it is optimally acceptable to SGMY in the United Kingdom. The salient issues previously identified include the following:

- There is insufficient SGMY-specific content in Rainbow SPARX [25,33]. In particular, the intervention did not satisfactorily address issues or topics of relevance for SGMY. For example, an SGMY research participant in the United Kingdom who had reviewed the intervention stated,“...You can’t just change a few words around and have a slightly different message at the start and say ‘oh yeah it’s a completely different game for LGBT [i.e. SGM] people’...” [33].
- The look and feel of Rainbow SPARX needs to be improved and updated [33]. A specific issue that needs to be addressed for SGMY is the forced sex binary inherent in the intervention (ie, the user can only customize a male or female avatar with no other options; see Figure 1 for details).
- The intervention should be made accessible across a range of platforms [33] so that it can be delivered on mobile phones, computers, and tablets.

**Figure 2:**
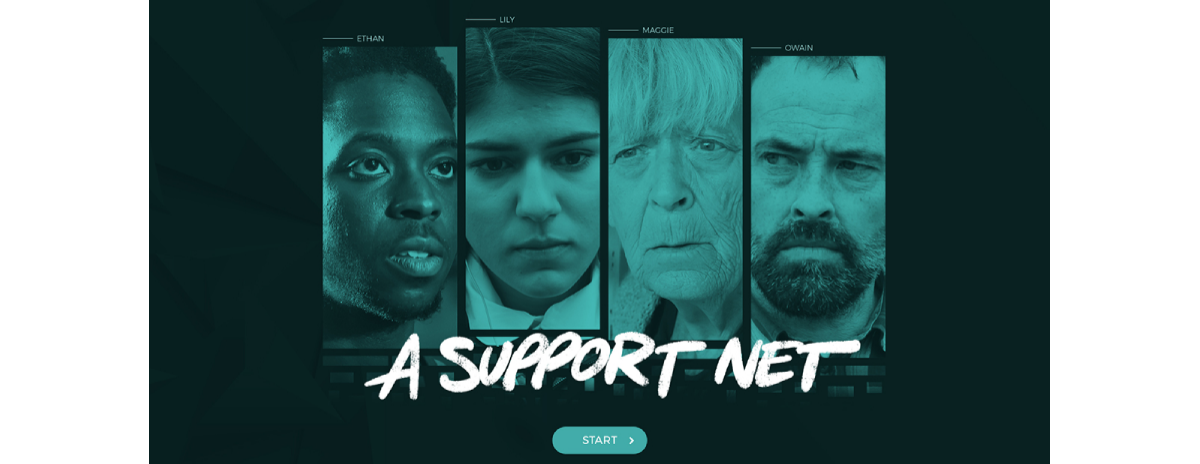
Landing page for *A Support Net* with the lead characters *Ethan*, *Lily*, *Maggie*, and *Owain* depicted.

Perhaps unsurprisingly then, when SGMY reviewing Rainbow SPARX (in its original format) in 2017 in the United Kingdom were asked if they would use the intervention if they were *feeling down*, only 38% (8/21) of participants indicated that they would do so [33]. This was among a sample for an exploratory qualitative study where 86% (18/21) of participants reported that they had felt down or low in the past (ie, they were ideally placed to assess an intervention for mild to moderate depression in SGMY) [33]. Given this feedback, we have decided that our new toolkit will not be an adaptation of an existing *mainstream* program such as SPARX, but instead it will be entirely *rainbow* in its focus. The PRIDE toolkit will be designed together with SGMY at the forefront, in an overdue effort to meaningfully center those who have been marginalized in terms of their mental health needs. Rainbow SPARX was designed with SGMY in New Zealand and is a fantasy-based serious game. However, consultation sessions with SGMY in the United Kingdom has indicated that they would prefer an intervention that reflects the United Kingdom’s contemporary context and is *real life* in terms of its look and feel (eg, as in A Support Net; Figure 2). Informed by earlier feedback from SGMY, we have codeveloped a new open educational web-based resource in a *real-life* style together with SGMY. This web-based resource is called “How to be a better LGBTQI+ [lesbian, gay, bisexual, trans, queer, intersex and other SGM persons] ally,” and it is delivered via the OpenLearn platform [34]. This recent work has helped inspire the toolkit development aspects of this study. However, despite the identified shortcomings of Rainbow SPARX, the intervention has meritorious aspects, as acknowledged previously by SGMY [25]. We are therefore keen to assess whether the new toolkit also provides self-help that is delivered in a novel format, has positive and likable *characters*, and offers helpful tips or strategies for coping [25].

### Underpinning Theoretical Concepts and Key Principles

The toolkit will be informed by several underpinning theoretical concepts and key principles, which will also support the design of the toolkit’s process evaluation and the parameters of a future effectiveness study.

#### CBT Principles

The general principles of CBT are the same for all groups, and CBT specifically for SGMY has been used effectively over a number of years (eg, [35,36]). However, CBT for SGMY requires some adaptation in order for the toolkit to adequately consider the unique challenges faced by these young people (eg, biphobia and the pervasiveness of cisheteronormativity).

#### Minority Stress

In the context of this project, we recognize that the mistreatment and high levels of stress that SGMY face place them at a greater risk of mental health problems [37]. It will therefore be explicit in the toolkit that we do not consider that SGMY are *lesser* or *more problematic* relative to their cisgender and heterosexual peers; rather, it is toxic social environments that place SGMY at elevated risk of mental ill-health.

#### Resilience

This is a person’s ability to *bounce back* in the face of adversity. Prior research has indicated that higher self-reported resilience is associated with lower levels of depression and anxiety [38]. Furthermore, resilience is thought to have a mitigating effect on depressive symptoms among people who have experienced challenging life events during childhood [38]. The aim of resiliency training in the toolkit will be to enhance an SGMY’s *bounce back*, resulting in the strongest possible capacity to recover from stressful events.

#### Co-design Principles

For this project, we will use the youth co-design participatory framework outlined by Hagen et al [30], an approach summarized for this project in further detail in Table 1 and the *Procedures* section.

#### Normalization Process Theory

This is a framework that can be used to describe and assess the implementation potential of complex interventions [39]. By using normalization process theory, we aim to think through the pertinent issues of implementation while designing the toolkit (eg, how it can fit within the practices of professionals supporting its use in local communities).

### Procedures

This project has 3 main objectives with 3 corresponding phases.

#### Phase 1

In the first phase, the objective is to co-design a media-rich evidence-informed web-based SGMY well-being prototype toolkit together with SGMY as well as key adult experts. In addition to the cocreation of contributions with SGMY at all stages of the project, there will also be input from key adult expert stakeholders. This will span the IDENTIFY, CONCEPT, and USE stages using the co-design stages or steps as outlined by Hagen et al [30] (see also Table 1 for an overview of all the stages and Table 2 for a summary in Gantt chart format). It is envisaged that the toolkit will include custom-made audiovisual materials in the form of dramatizations to illustrate key points from the vignettes created during the POSITION and CONCEPT stages, for example, dramatizations in a style as adopted in *A Support Net*—a free mental health literacy resource with >30,000 users to date developed by PRIDE team members and delivered via the Open University’s OpenLearn platform [40] (Figure 2). There will also be components specifically designed to enhance engagement as recommended by SGMY during the CONCEPT and CREATE stages (eg, a points-based rewards system).

**Table 2:** Summary Gantt Chart for the study.

Milestones	Before the study starts				Study month																	
Project Month				1		2	3	4	5	6	7	8	9	10	11	12	13	14	15	16	17	18
Milestone 1: ethics approval sought and obtained	✓	✓	✓																			
Milestone 2: appoint 2 advisory groups		✓	✓																			
Milestone 3: phase 1 (step 1) complete scoping review				✓		✓	✓	✓														
Milestone 4: phase 1 (step 1) SGMY^a^ interviews or focus groups						✓	✓	✓														
Milestone 5: phase 1 (step 1) interviews with adult experts						✓	✓	✓														
Milestone 6: phase 1 (Step 1) confirm theoretical framework				✓		✓	✓	✓	✓													
Milestone 7: phase 1 (step 2) 1st SGMY co-design workshops										✓	✓											
Milestone 8: phase 1 (Step 2) dramatizations filmed & prototype ready												✓	✓									
Milestone 9: phase 1 (Step 2) 2nd SGMY co-design workshops														✓	✓							
Milestone 10: phase 2 Refined prototype ready for evaluation																✓	✓					
Milestone 11: phase 2 Confirm assessments for evaluation														✓	✓	✓						
Milestone 12: phase 3 Process evaluation completed																		✓	✓	✓		
Milestone 13: phase 3 Interviews with SGMY & adult experts																			✓	✓		
Milestone 14: phase 3 draft initial protocol for future trial																			✓	✓		
Milestone 15: create summary report for SGMY and for adult experts																				✓	✓	✓
Milestone 16: create academic outputs																				✓	✓	✓
Advisory group meetings (×3)				✓						✓									✓			

^a^SGMY: sexual and gender minority youth.

The novel web-based toolkit will be underpinned by a new framework of useful and evidence-informed cognitive and behavioral coping strategies. The framework will draw upon practice wisdom from expert practitioners in SGMY mental well-being (in the IDENTIFY stage), from the *expert by experience* solutions offered by SGMY (in the DEFINE stage), and from the scoping review, which will appraise the peer-reviewed literature (also in the DEFINE stage). For instance, content is likely to include evidence-informed strategies to improve a young person’s psychological and physical health (eg, behavioral activation challenges involving physical exercise as has been previously employed in Rainbow SPARX [21]).

To draw upon the experiences of a range of SGMY from diverse backgrounds, the co-design workshops are planned to be on the web with participants from SGMY organizations (where many members are Black, Asian, or from another minority ethnic group). Ideally, some adolescents questioning their sexuality or gender identity will take part in this project. However, if they are *currently unsure*, these youth are less likely to participate, which is an issue also identified in earlier research [41]. Hence, while we will proactively attempt to recruit questioning youth, we will also ask all SGMY participants to reflect on their early experiences of *coming out* in developing the intervention so that we surface issues of salience for questioning adolescents. Composite vignettes for the audiovisual materials and the related scenarios will be developed with SGMY (during the POSITION to CREATE stages).

The key activities in phase 1 will occur in 2 concurrent steps.

*Step 1* will consist of interviews with SGMY and adult experts as well as a scoping review focused on identifying adaptive psychosocial coping strategies for SGMY (all conducted during the IDENTIFY and DEFINE stages of the study). The four key activities will be as follows:

1. Informed by an earlier scoping review and subsequent conceptual framework from Colpitts and Gahagan [42,43], we will conduct a scoping review that describes the challenges in SGMY’s social and environmental systems and establishes the recommended strategies for building resilience. In addition, the measures used in the studies identified during the scoping review will be assessed for their suitability for use during the process evaluation of the toolkit in phase 3.
2. Informed by our prior work, advice from practitioners with expertise in cognitive and behavioral coping strategies for SGM individuals, as well as other contributions including those from the scoping review, we will develop a framework of the components and behavior change techniques required to enhance resilience in SGMY.
3. We will conduct web-based (or face-to-face, if feasible) interviews (or focus groups—depending on participants’ preferences) with SGMY focusing on the common environmental problems SGMY experience and the associated stress management strategies frequently used by SGMY (n=10).
4. We will conduct web-based (or face-to-face, if feasible) interviews with adult experts to ascertain how the web-based toolkit could be used in community settings and public health systems (as well as by SGMY), including, for example by, (1) health and social care commissioners (n=5); (2) parents of adolescents supportive of SGMY (n=5); and (3) practitioners (eg, SGM youth workers, school pastoral care workers, police officers, child and adolescent mental health services clinicians; n=5). The interviews with adult experts will also include determining adaptive resilience and stress management strategies for SGMY.

*Step 2* will involve co-designing the toolkit (for work across the POSITION, CONCEPT, and CREATE stages). The key activity will be working collaboratively with SGMY (n=20) during at least two sessions to cocreate the web-based toolkit at participatory web-based co-design workshops. SGMY user requirements and preferences will be used to inform the work of the contracted information technology specialists, so that they can produce the prototype toolkit. Audiovisual material will be developed based on vignettes created together with SGMY.

#### Phase 2

The second main objective of this project is to explore how the toolkit can be used within public health systems in the United Kingdom by SGMY. We will establish how the toolkit could be used by SGMY and potentially by others (eg, health and social care professionals). For example, the toolkit may also be useful for professionals’ future continuing professional development activities.

The two key activities in phase 2 during the CONCEPT and CREATE stages are as follows:

1. Completing the initial build of the prototype toolkit and refining this as needed based on SGMY feedback (n=20, ie, the SGMY participants from phase 1, step 2)
2. Determining SGMYs’ (n=20) and adult experts’ (n=15, ie, from phase 1, step 1) user-defined key criteria for success (eg, SGMY may determine that the toolkit’s engagement potential is especially important) and potentially the standardized assessments for the toolkit (eg, the measures to be used)

#### Phase 3

The third main objective of this project is to plan the potential delivery of the intervention and determine the design and measures for a future effectiveness study, as well as further implementation of the toolkit. Assuming that *stop/go* decision criteria to determine if the toolkit warrants an effectiveness study are met (criteria developed during the CREATE stage) and based on the in-depth process evaluation conducted in the USE stage, we will submit a bid for an effectiveness randomized controlled trial to the National Institute for Health Research in the United Kingdom (or another relevant funder).

The key activities in phase 3 during the USE stage are as follows:

1. An in-depth process evaluation will be conducted with (n=10) SGMY not involved in the IDENTIFY to CREATE stages of the project. Data will then be gathered directly from the prototype toolkit (eg, the psychological measures data) and during recorded interviews. During interviews, SGMY will be asked to think out loud while interacting with the toolkit, as informed by an earlier evaluation conducted with young sexual minority men by Fleming et al [23]. Therefore, SGMY participants will use the toolkit in a time-bound lab-based session (eg, during 2 mornings of possible use) where SGMY may elect to only use the PRIDE toolkit for a much shorter period (eg, 40 minutes each morning). This will yield less in the way of potential real-world use data, but given the think out loud methodology, this is a pragmatic approach allowing for some limited use data to be obtained.
2. Interviews will also be carried out with selected adult experts, including commissioners of health and social care services, to establish the feasibility of implementation at scale, to confirm outcome measures, and to determine how the toolkit can be best used in public health systems (n=5). The interviews will help inform the design of a possible future effectiveness study.

### Measures

The measures to be embedded into the actual toolkit will be determined during phase 1 of the project. Indicative measures include assessments previously used in evaluating Rainbow SPARX, such as the following:

- The Kazdin Hopelessness Scale for Children, which is a 17-item self-report questionnaire assessing hopelessness [44]
- The Mood and Feelings Questionnaire, which is a 33-item self-report questionnaire designed to detect depression in clinical populations [45]
- The Pediatric Quality of Life and Satisfaction Questionnaire, which is a 15-item self-report questionnaire addressing satisfaction with current life [46]

Other options that are suitable for adolescents and focus on well-being are as follows:

- The World Health Organization-5 Well-being Index, which is a 5-item measure of overall well-being [47]
- The University of California Los Angeles Loneliness Scale for Adolescents, which is a 20-item self-report measure [48]
- The Warwick-Edinburgh Mental Well-being Scale, which is a 14-item positive mental health measure [49]

### Proposed Analyses

The analyses will be carried out with regard to the 3 main objectives of the project in the following ways.

#### Objective 1

Together with *SGMY* as well as key adult experts, we aim to co-design a media-rich evidence-informed web-based *SGMY* well-being prototype toolkit aimed at those aged 13 to 19 years*.* The data pertaining to this objective will include interviews, focus groups, and a scoping review. We will use a general inductive approach for data analysis of transcribed interviews and focus groups [50,51]. The summarized results from the scoping review, together with the themes and subthemes derived from the interviews and focus groups, will lead to the creation of a novel and timely theoretical framework for evidence-informed cognitive and behavioral coping strategies for SGMY. We anticipate that this framework will be relevant to others working to support the mental health of SGMY in the United Kingdom and further afield.

#### Objective 2

We will explore how the web-based toolkit can be used within public health systems in the UK by *SGMY* and potentially other relevant stakeholders*.* The data related to this objective consist of transcribed interviews. These transcripts will be analyzed using the framework approach [52], an approach that is closely related to established methods (eg, thematic analysis) and has already been applied in earlier health-related research (eg, [53]). For the PRIDE study, facets of SGMY stressor experiences and exposures will be identified via findings from the existing literature (as part of objective 1). Framework analysis allows for the use of this prior knowledge to gain further depth to a phenomenon; this is done through exploring shared communalities and differences in experiences within the participant group. In this study, we assume there will be areas of tension in which experiences are qualitatively different for SGMY participants, by virtue of their membership in a particular subgroup or social identity (eg, sexual minority vs gender minority differences). Unlike some qualitative analytic methods that engender homogeneity in sampling to explore the aims (eg, interpretative phenomenological analysis) [54], framework analysis is well suited to analyses conducted with heterogeneous participant groups. Thus, participants’ shared experiences, as well as diversity of experiences, can be explored [55]. The approach involves working through stages to develop themes (as with thematic analysis), but then the themes are further refined in an iterative fashion, eventually leading to the development of a robust conceptual framework. The analyses pertaining to implementation are guided by the normalization process theory [39]. In addition to the themes and subthemes identified from the interviews, recommendations for future plans for the toolkit will be developed with SGMY and stakeholders to build into the project opportunities for novel and unforeseen ways to use and access the toolkit.

#### Objective 3

We aim to conduct a preliminary evaluation of the acceptability, feasibility, and impact of the toolkit, which will inform the design of a future effectiveness study. Semistructured interviews using the *think out loud* methodology will be conducted. These will include questions related to the following: participants’ views about the acceptability of the toolkit; the extent to which the toolkit adequately addresses the specific needs of SGMY, including those with diverse characteristics (eg, in regard to the user-generated criteria for the toolkit’s perceived *success*); areas for improvement; the feasibility of using it in different real-world contexts; and the possible impacts of the toolkit on users. The transcribed interviews will be analyzed using the general inductive approach [50,51]. Preliminary use data will also be examined, including the time spent using the toolkit and the extent to which different components were used. Preliminary information about the potential impact of the intervention will be gained by examining the effect sizes of pre- to postintervention use changes in the primary outcome measure (to be determined) and potentially other self-report measures.

### Study Team

The PRIDE team consists of a project coordinator and researchers with expertise in youth mental health, SGM well-being, intervention development and implementation, and public health research. This team will work with 2 advisory groups. One advisory group will consist of at least four SGMY advisors. The second advisory group comprises adult experts, including academics and others, with practice and policy expertise in areas such as e-therapies, SGM public health, and SGM youth work. Both advisory groups will be involved from project initiation to completion, including the planning of the subsequent effectiveness study (which is beyond the scope of this protocol).

### Ethics and Consent

A favorable opinion from the Open University’s human research ethics committee will be obtained before participants are recruited for this study. SGMY and key adult experts participating in this study will provide written consent before participating. The consent form and participant information sheet for SGMY participants will be reviewed by SGMY and refined to ensure that it is presented in a way that is age-appropriate and easy to understand. It will also contain the contact details for SGMY supports (eg, telephone-based helplines) and suggestions on how to seek SGMY-appropriate psychosocial support if required.

Involving adolescents interested in this study who are aged <16 years are likely to present ethical challenges, because these younger adolescents will be required to have written consent from a parent or legal guardian (as well as their own written consent). This presents problems for younger SGMY who are not yet *out* regarding their SGMY status. Therefore, only those younger SGMY with the coconsent of a parent or legal guardian can participate. As a result, the sample of SGMY is likely to be skewed toward older adolescents in this study, and those who are younger will already be *out* with supportive families.

## Results

This study was funded in March 2021. We received a favorable opinion for our ethics application for phase 1 of the study from the Open University’s human research ethics committee in August 2021. We will apply for further approval for phases 2 and 3. The study commenced on September 1, 2021. Participant recruitment for the study began in October 2021, and all data for the study should be gathered by early 2023 (see Table 2 for details).

## Discussion

### Principal Findings

There has been considerable research describing the mental health challenges SGMY face, but little research has been conducted on interventions designed to make a difference for SGMY. For example, 2 prior systematic reviews of psychosocial treatments have previously identified only a single evaluated digital tool to support the mental well-being of SGMY [19,20], and a third more recent and inclusive systematic review highlighted only 5 interventions that broadly targeted mental health–related problems [22]. Fortunately, this situation is rapidly changing, with a recent research protocol highlighting an emerging interest in gamified health interventions for SGM individuals [56]. For example, an intervention is being pilot-tested among SGMY (aged 14-18 years) in the United States, with the aim of improving help-seeking behavior and coping [57]. Our proposed toolkit is therefore timely and could be useful in the United Kingdom, where there is an absence of evidence-informed digital interventions to support the well-being of SGMY. This project is funded by the UK’s Medical Research Council under the Public Health Intervention Development scheme. This scheme involves developing a novel intervention, and coproduction with relevant stakeholders is expected. Within the rules of the scheme, limited resources (approximately 15% of the project’s overall costs) are directed toward acceptability and feasibility research. Therefore, the emphasis of this project is directed toward creating a new toolkit (during phases 1 and 2), with only a preliminary emphasis placed on evaluation (in phase 3).

The focus of our work, and the limited earlier intervention research focused on SGMY in this field, has been directed at an individual level. For instance, *Rainbow SPARX* [21] and *AFFIRM Online* [26] have sought to reduce symptomatology, such as depressive symptoms, among individual SGMY. However, there are wider systemic issues that require attention in the United Kingdom and elsewhere, because SGMY are forced to live in a challenging heteropatriarchal society. As a result, SGMY are frequently *problematized*, whereas insufficient effort is directed at addressing the negative environments that mean SGMY are more likely to have mental health problems. In short, more research should be conducted to improve the overall milieu for SGMY. This is because earlier work, drawing on population-based research, has already demonstrated that schools that are more supportive of SGMY lead to a reduction in risk in terms of depression and suicidality for SGMY [6]. Although it is envisaged that our toolkit will focus on individual SGMY, it could also potentially be used by others (eg, health and social care professionals) for their continuing professional development. Thus, the toolkit could reach a *wider audience* that might also assist in helping change certain environments.

Our decision to focus on the larger (heterogeneous) SGMY population versus being more specifically focused on either sexual minority or gender minority youth is a limitation of this study, given some of the challenges unique to each of these 2 main groups. However, the use of a framework analytic method for qualitative data interpretation allows for the exploration of group commonalities and differences [55], which might mitigate some of the limitations related to being able to identify key differences between subgroups in the overall SGMY sample. As a consequence, our intention is to cocreate a toolkit that will be meaningful and acceptable to both sexual as well as gender minority youth. In addition to drawing on shared experiences of stressors SGMY face, our qualitative work will also explore areas of tension, in which gender and sexual minority young people discuss issues that they perceive differentiate SGMY subgroups, for example, specific stressors that vary along group divides. We will attempt to recruit a range of SGMY participants and include those who live outside of the largest cities in the United Kingdom, but a probable limitation of this study will be an overrepresentation of SGMY participants from *LGBTQ+ (lesbian, gay, bisexual, trans, queer) friendly* urban areas. Our relatively short timescale poses other challenges for both the intervention and its evaluation, as well as its possible real-world implementation [58]. This is because, for digital toolkits, technology is evolving rapidly and interventions that are not updated (or refined following trials) risk becoming dated even before an eventual roll-out [59]. Consequently, a *new* toolkit could become much less appealing to SGMY by the time it is available outside of research settings. Another challenge in this rapidly changing field is that interventions are required to keep pace with technology (eg, work across a range of devices and operating systems) while also demonstrating effectiveness. These challenges have already been reinforced as key issues in relation to digital interventions for young people [58].

### Conclusions

SGMY are underserved in terms of mental health service provision, and these young people have already highlighted the value of support on the web [33]. Our proposed project seeks to coproduce an engaging media-rich evidence-informed well-being prototype toolkit for this unique subpopulation. SGMY will be central to the creation of the toolkit, which will require an effectiveness trial after this initial work is completed.

## Abbreviations

CBT: cognitive behavioral therapy
LGBTQ+: lesbian, gay, bisexual, trans, queer and other sexual and gender minority persons
LGBTQI+: lesbian, gay, bisexual, trans, queer, intersex and other sexual and gender minority persons
PRIDE: Promoting Resilience and Well-being Through Co-design
SGM: sexual and gender minority
SGMY: sexual and gender minority youth
SPARX: Smart, Positive, Active, Realistic, X-factor thoughts
